# From the Editor’s Desk: the Evolving Journey of Engaging Patients as Research Partners

**DOI:** 10.1007/s11606-021-07315-1

**Published:** 2022-03-29

**Authors:** Dominick L. Frosch

**Affiliations:** Health Science Diligence Advisors, LLC, Redwood City, CA USA

When the US Congress passed the Affordable Care Act in 2010, it also created the Patient Centered Outcomes Research Institute (PCORI), which was reauthorized for 10 years in 2019. The creation of PCORI not only made available new opportunities to fund and support comparative effectiveness research and studies focused on improving patient-centered outcomes, but also introduced a new approach to conducting research by requiring investigators to actively engage patients and other relevant stakeholders not just as study participants but as active partners in the research process—from inception of an idea all the way through to the conclusion of project activities and communication and dissemination of results. Similarly, the Veterans Affairs Department of Health Services Research and Development (VA HSR&D) has embraced the concept of partnered research, in alignment with the goals of the learning health system, to improve and accelerate the uptake of research innovations and discoveries in clinical practice. This special supplement of *JGIM* on patient and veteran engagement in research is funded jointly by PCORI and VA HSR&D with the goal of sharing lessons learned from partnered research with the broader field of health services research and health care delivery science researchers.

The response from the research community to the call for papers for this special supplement was very robust with a total of 82 submissions. Of these, 12 were judged to not be responsive to the call for papers and were therefore considered for publication in *JGIM* at large. Of the remaining 70 submissions that were deemed responsive, 27 were reports of original research, 20 were perspectives, and 23 were narratives. From these, the editorial team for the supplement, with the support of *JGIM’s* excellent peer reviewers, selected 21 papers that are included in the issue, providing a rich reflection of how partnering with patients and veterans in research has evolved over the past decade.

In partnering with PCORI and VA HSR&D, *JGIM* agreed to try a new category of papers—narratives—that has not previously been something the journal has considered. Our goal was that narratives would help provide a more complete picture of the lessons learned from engaging patients and veterans in the research process, lessons that can be hard to describe and quantify with existing research tools, as well as nuances of experiences with the research process that are easily missed or lost in original research papers and perspectives that build on existing theories and evidence.

The narratives we received came in a variety of forms, but contrary to our hope only a minority were first person accounts from patients and veterans describing their experiences partnering with researchers. Others were collaborative efforts from patients, veterans, and researchers, describing their shared experiences and lessons learned of how to work together most effectively in solving the many challenges that will inevitably arise in any research project^1–5^. In light of our own inexperience with this article category, we did not provide any specific guidance to peer reviewers for the narratives they reviewed. Despite this, our peer reviewers, overall, embraced this new category of articles and provided overwhelmingly constructive feedback to authors. One clear lesson learned, which is especially relevant for narratives written solely by patients or veterans, is that care is needed when making expansive judgements about the state of a research field. Peer reviewers usually objected to such statements. Judging the state of a field can be a minefield, even for researchers, and we clearly should have guided patients to focus specifically on their personal experiences. A broader lesson for *JGIM* is that future solicitations of narratives will perhaps require more detailed guidance to help a greater number of potential patient authors frame their experiences as stories to share with the research community and gain confidence to tell their personal stories in this format.

Nevertheless, several clear themes emerge from the narratives included in this supplement about what effective engagement of patients and veterans as research partners requires. Just like when patients receive clinical care, the experience of being respected by the research team is critical to the successful engagement of patients and veterans as partners in the research process^6^. Joining a research team often feels intimidating for patient and veteran partners. Consistently conveying a sense of respect is what empowers partners to make the valuable contributions they can and do make. But a critical part of respecting these new partners is to also provide training—to recognize that research has a language of its own that is foreign to someone without years of formal training. That this foreign language is spoken by people with many letters and degrees after their name only adds to the sense of intimidation felt by patient and veteran partners.

Respecting and training patient and veteran partners brings benefits for researchers as well as the new partners. Research becomes more relevant because patients can provide researchers with a richer more complete picture of their experience of illness and receiving care for it. Patient partners can point researchers to critical aspects of the illness experience that researchers hadn’t trained their lens to observe or ameliorate. Moreover, patient partners often bring other assets beyond their own illness experience to a research team—their professional skills and experience from their own careers. Research teams that leverage these assets are bound to be more successful. Patient partners in turn are proud to do their part to contribute to the never-ending process of improving the care while also experiencing personal and career growth in ways they hadn’t anticipated.

Shifting the paradigm of how research is conducted—engaging patients and veterans as active partners from inception to dissemination—requires a cultural change. In our current technological age, everyone is looking for ideas and concepts that are scalable. But a little discussed reality is that some things are very difficult to scale, if they can be scaled at all, in the network sense of the term. The narratives included in this special supplement clarify the hard work that we need to do in continuing to change the culture of research—away from an exclusive process driven by experts to an inclusive process that recognizes the value of multiple perspective and voices—and also helps us more clearly see the range of benefits that accrue from this shift. To use PCORI’s words, research done differently is research done better and as researchers that should be our overarching goal.
